# Intake of added sugar, fruits, vegetables, and legumes of Portuguese preschool children: Baseline data from SmartFeeding4Kids randomized controlled trial participants

**DOI:** 10.3389/fnut.2023.1150627

**Published:** 2023-03-29

**Authors:** Sofia Charneca, Ana Isabel Gomes, Diogo Branco, Tiago Guerreiro, Luísa Barros, Joana Sousa

**Affiliations:** ^1^Laboratório de Nutrição, Faculdade de Medicina, Centro Académico de Medicina de Lisboa, Universidade de Lisboa, Lisbon, Portugal; ^2^Research Center for Psychological Science (CICPSI), Faculdade de Psicologia, Universidade de Lisboa, Lisbon, Portugal; ^3^LASIGE, Faculdade de Ciências, Universidade de Lisboa, Lisbon, Portugal; ^4^Instituto de Saúde Ambiental (ISAMB), Faculdade de Medicina, Centro Académico de Medicina de Lisboa, Universidade de Lisboa, Lisbon, Portugal

**Keywords:** preschool children, dietary intake, added sugar, fruits, vegetable, legumes

## Abstract

**Introduction:**

The SmartFeeding4Kids (SF4K) program is an online self-guided intervention for parents with the propose of changing parental feeding practices and children’s dietary intake, focusing on the intake of added sugars, fruit, vegetables, and legumes. This paper aims to describe children’s dietary pattern at baseline through a 24-h food recall, the SmartKidsDiet24.

**Methods:**

Overall, 89 participants recorded at least one meal of the 3-day food recall. Mean age was 36.22 ± 6.05 years and 53.09 ± 15.42 months old for parents and children, respectively. Of these, 22 participants were considered to have 2 days of near complete 24-h food recalls. Children’s dietary intake are reported for these 22 participants based on parents reports and, thus, represent estimations only, as it remains unknown whether children consumed other non-reported foods.

**Results:**

Fruit was the group with the highest daily intake among children (mean 1.77 ± 1.10 portions/day), followed by added sugar foods (mean 1.48 ± 0.89 portions/day), vegetables [median 1.27 (1.64) portions/day] and legumes [median 0.12 (0.39) portions/day]. Fruit intake was positively correlated with vegetable intake (*p* = 0.008). Regarding Dietary Reference Values accomplishment, 13.6% of children exceeded the daily safe and adequate intake of sodium, 77.3% did not meet potassium and fiber recommendations, and 31.8% did not meet vitamin C recommendations.

**Discussion:**

All children did not meet calcium, vitamin B12 and vitamin D intake recommendations. Our findings further justify the need for dietary interventions in this field, to improve young children’s diets.

**Clinical trial registration:**

ClinicalTrials.gov, identifier NCT04591496.

## 1. Introduction

Childhood obesity has become one of the most challenging public health crises of our time (1, 2). Overall, the worldwide proportion of overweight children and adolescents aged 5–19 years old rose from 1 in 10 to almost 1 in 5 from 2000 to 2016 (3). Nationally, 29.7% of Portuguese children aged 6–8 years old were overweight, and 11.9% were obese (4). The key drivers of this increase in the prevalence of overweight include changing dietary patterns and is accompanied by short-term and long-term consequences, such as an increased risk of developing non-communicable diseases later in life (5). Moreover, the majority of children that are overweight tend to remain in the same Body Mass Index (BMI) category during their adult life, resulting in a significant economic burden on society (6). Hence, early interventions to target childhood obesity are warranted. Mobile health technologies, including mobile apps for weight management, to improve nutrition behaviors and nutrition-related health outcomes are becoming more popular than ever and are reported to represent a highly promising approach for combating childhood obesity and/or inadequate eating patterns (7, 8). We have changed our dietary patterns, leaving behind traditional diets and consuming foods that are frequently low in essential nutrients and fiber, and high in fats and sugars (3, 9). While there is no nutritional requirement for free sugars, the consumption of sugars in the European pediatric population exceeds current recommendations (10). Contrary to a Western style diet, healthier alternatives are higher in plant-based foods, including fresh fruits, vegetables, and legumes (11). Portuguese children and adolescents have been shown to have a higher inadequacy of fruit and vegetable consumption when compared with adults and the elderly (12). This highlights the need to address the intake of these key food groups among the youth. The SmartFeeding4Kids (SF4K) program was developed. This paper aims to describe the children’s dietary pattern at baseline of the SmartFeeding4Kids (SF4K) program, focusing on the intake of added sugars, fruits, vegetables, and legumes (13).

## 2. Materials and methods

### 2.1. Study design and population

The SF4K randomized controlled trial (RCT) study protocol is described in detail elsewhere (13), namely the characteristics of the intervention, study design, procedures and outcome measures. The SF4K program is an online self-guided intervention for parents with the propose of changing parental feeding practices and children’s food intake, focusing on the intake of added sugars, fruit, vegetables, and legumes. The intervention intends to promote positive changes in parental feeding practices and their children’s diet through self-regulation strategies and other behavior change techniques (13). The study population was Portuguese preschool children (2–6 years old). The study was approved by the Ethics and Deontology Committee of the Faculty of Psychology, University of Lisbon. Recruitment was conducted nationally, being open to all parents of 2 to 6-year-old children living in Portugal who wanted to participate in this study. Social networks and online groups attended by parents were used to promote and share this trial. Eligibility criteria were being fluent in Portuguese, a parent/caregiver of one 2- to 6-year-old child at baseline and have a mobile phone or computer/tablet with internet access (13). Once parents completed the registration on the SF4K app, they were invited to answer a baseline assessment protocol, including demographic information, parent’s and children’s weight and height, age and gender, parent’s educational level, kinship with the child, number of children and adults in the household, if parents receive child benefits, birth date, childcare attendance and food intolerances and allergies. Baseline assessment also included recordings of their child’s food and portion intake for 3 days (two weekdays and one weekend day), though a 24-h food-recall. Both data from the SF4K (intervention group) and psychoeducational control condition groups were included in this analysis.

### 2.2. Data collection

Data was collected from July 2021 to May 2022. An online 24-h food recall that uses the electronic food composition database by INSA (National Institute of Health Doutor Ricardo Jorge) was developed for this study, theSmartKidsDiet24, where parents recorded all the foods they are sure that their children ate (could be foods prepared by parents or eaten in their presence) in the specific days chosen by the app. Of interest, the database was updated with sugar-sweetened foods/beverages and other processed foods frequent in Portuguese children’s diets, as well as vegetarian/vegan alternatives. Parents were guided on adequate measurement of food portions with the child’s hand. Foods eaten without the presence/supervision of parents, were not recorded. Therefore, most children did not have fully completed food recalls. As we cannot truly quantify dietary intake without the recording of a full day of eating, we focused this analysis only on the participants that had recorded a total of 5 main meals (breakfast, morning snack, lunch, afternoon snack, and dinner) in at least 2 days of the 3-day 24-h recall. Considering children with at least 2 complete 24-h recall’s, mean dietary intake of added sugar foods, fruits, vegetables, and legumes were calculated per meal. For each of these 4 food groups, portions and the following macro and micronutrients were analyzed: energy, protein, carbohydrates, monosaccharides and disaccharides, fiber, total fats, monounsaturated fats, polyunsaturated fats, calcium, potassium, sodium, vitamin B12, vitamin C, and vitamin D. The total daily intake of each of these nutrients was calculated for each food group. The referred micronutrients were chosen, as added sugar foods consumed by Portuguese children are often dairy products, frequently fortified with vitamin B12 and/or vitamin D (e.g., chocolate/flavored milk, yogurt, and ice-cream), hence the evaluation of calcium, vitamin 12 and vitamin D. As foods with added sugar are often processed foods with high sodium content, this micronutrient was also assessed. Since fruits and vegetables are the main sources of dietary potassium and vitamin C, they were also detailed in this paper. To assess whether children met their nutrient requirements, Dietary reference values (DRVs) were collected from the DRV Finder Tool by the European Food Safety Authority, EFSA (14). The average requirement (AR) was considered for energy (kcal), calcium (mg), and vitamin C (mg). The adequate intake (AI) was considered for fiber (g), potassium (mg), vitamin B12 (μg), vitamin D (μg). Safe and adequate intake was used for sodium (g). Children’s and parents’ BMI were calculated as weight/height squared (kg/m^2^), based on parent’s report on their and their children’s height and weight. Z-scores of the child’s weight, height and BMI for age and sex were calculated using the WHO AnthroPlus software (15). According to BMI, parents were classified as follows: underweight (<18.5 kg/m^2^), normal weight (18.5–24.9 kg/m^2^), pre-obese (25–29.9 kg/m^2^), and obese (≥30.0kg/m^2^).

### 2.3. Data analysis

Statistical analysis was conducted using SPSS version 27.0 (SPSS^®^ Inc., Chicago, IL, USA). Depending on the sample size, the Normal distribution of the variables was verified using the Kolmogorov–Smirnov or Shapiro–Wilk tests. Data from categorical variables were described as frequencies (percentages). Normally distributed continuous variables are presented as mean ± standard deviation (SD). The median (interquartile range) was presented when data from continuous variables were not normally distributed. The hypotheses and statistical analysis were specified before the data were collected. Groups were created based on parental reporting of the 24-h food recall, theSmartKidsDiet24 (high reporting: recorded a total of five meals in at least 2 days of the 3-day 24-h food recall; low reporting: did not record five meals in at least 2 days of the 3-day 24-h food recall). Whole-day dietary intake estimations were calculated for participants with a high reporting on theSmartKidsDiet24 only, by simply averaging the intake of the days with the recording of at least five main meals. Average values were calculated considering 3 days, for participants with recordings of five main meals in a total of 3 days, or considering 2 days, for participants with recordings of five main meals for only 2 days. Reported per meal data also represents averaged data from the referred recorded days. Comparisons between groups were performed using the Students *t*-test for normally distributed data. The non-parametric alternative (Mann–Whitney) was used when data were not normally distributed. Chi-squared test was used for comparisons between categorical variables, and Chi-squared test by Monte Carlo simulation was used when the conditions for the chi-squared test were not met. Fisher’s exact test was used in the analysis of contingency tables. A *p*-value of less than 0.05 was considered statistically significant.

## 3. Results

Overall, 89 participants recorded at least one meal of the 3-day 24-h food recall at baseline. Our sample was constituted mostly by mothers (93.26%, *n* = 83) and sons (56.18%, *n* = 50). The mean age was 36.22 ± 6.05 years and 53.09 ± 15.42 months old for parents and children, respectively. Most children’s household included both parents (43.82%, *n* = 39) or both parents and sibling(s) (43.82%, *n* = 39). Regarding education levels, more than half of parents had a university degree (60.67%, *n* = 54). Of these 89 participants, 9 recorded a total of five meals for each of the 3 days and 13 recorded five meals in 2 days, resulting in a total of 22 participants with a high reporting to the theSmartKidsDiet24 at baseline. The meal with the most recordings was breakfast, followed by dinner, afternoon snack and lunch. Morning snack, night snack and extras were the meals with the least recordings. Table 1 shows the number of recordings for each meal of the 3-day 24-h food recalls. Table 2 details children’s and parents’ characteristics of the whole sample (*n* = 89), and according to reporting to theSmartKidsDiet24. Of the 22 children with a total of 5 main meals in at least 2 days, 54, 55% (*n* = 12) were girls and the mean age was 54.30 ± 17.12 months. No significant differences were found between groups of reporting to the theSmartKidsDiet24 regarding children’s age categories (*p* = 0.829), age (*p* = 0.673), gender (*p* = 0.243), weight for age Z-score (*p* = 0.244), height for age Z-score (*p* = 0.417), BMI for age Z-score (*p* = 0.499), household (*p* = 0.499), food intolerances (*p* = 1.000), parent’s age (*p* = 0.123), parents education level (*p* = 0.488), parents BMI (*p* = 0.281), or parents nutritional status according to BMI (*p* = 0.751). Regarding the dietary intake of the four food groups evaluated in the sample with high reporting to the theSmartKidsDiet24, fruit represented the group with the highest daily intake among children (mean 1.77 ± 1.10 portions per day), followed by added sugar foods (mean 1.48 ± 0.89 portions per day), vegetables [median 1.27 (1.64) portions per day] and lastly by legumes [median 0.12 (0.39) portions per day]. The caloric intake of monosaccharides and disaccharides (sugar) from added sugar foods was above 10% of daily energy requirements in most children (81.8%). Detailed whole-day dietary intake of macro and micronutrients from each food group according to children’s age is shown in Table 3. Dietary intake of macro and micronutrients from each food group discriminated by meal and according to children’s age are shown in Tables 4–7. Daily fruit intake was positively correlated with daily intake of vegetables (*p* = 0.008). Children’s BMI for age Z-score was positively associated with daily intake of vegetables (*p* = 0.007), as well as the energy (*p* = 0.043), protein (*p* = 0.007), carbohydrates (*p* = 0.048), monosaccharides and disaccharides (*p* = 0.003), fiber (*p* = 0.007), and polyunsaturated fatty acids (*p* = 0.007) prevenient from vegetables. No associations were found for other food groups regarding children’s BMI for age Z-score. Parents BMI and education level were not associated with children’s intake of these four food groups. Children’s age was positively correlated with daily energy (*p* = 0.029), monosaccharides and disaccharides (*p* = 0.003), fats (*p* = 0.018) and saturated fats (*p* = 0.034) intake from added sugar foods, but not from fruit, vegetables, or legumes. Regarding children’s DRV accomplishment, 13.6% of children exceeded the daily safe and adequate intake of sodium, 77.3% did not meet potassium and fiber AI, and 31.8% did not meet vitamin C AR. Moreover, 100% of children did not meet calcium AR, vitamin B12 and vitamin D AI, considering nutrient intake from the food groups evaluated.

**TABLE 1:** Number of recordings for each meal of the 3-day 24-h food recalls out of all 89 participants.

Meal/day	Day 1	Day 2	Day 3	Total recordings per meal
Breakfast	83	57	50	190
Morning snack	39	30	20	89
Lunch	43	55	24	122
Afternoon snack	48	47	36	131
Dinner	62	51	43	156
Night snack	25	15	10	50
Extra meal	13	9	8	30

**TABLE 2:** Overall children’s and parent’s characteristics and according to reporting to theSmartKidsDiet24.

	Whole sample (*n* = 89)	Low reporting to theSmartKidsDiet24 (*n* = 67)	High reporting to theSmartKidsDiet24 (*n* = 22)	*p*-value
**Child’s age**				
≤3 years old [*n* (%)]	30 (34)	23 (34)	7 (32)	0.83^a^
≥4 years old [*n* (%)]	59 (66)	44 (66)	15 (68)	
Age (months), mean ± SD	53.1 ± 15.4	52.7 ± 14.9	54.3 ± 17.1	0.67^b^
Age (years), mean ± SD	4.4 ± 1.3	4.4 ± 1.3	4.5 ± 1.4	0.67^b^
**Child’s gender**				
Girl [*n* (%)]	39 (44)	27 (40)	12 (55)	0.24^a^
Boy [*n* (%)]	50 (56)	40 (60)	10 (45)	
**Child’s growth standards**				
Weight for age Z-score, median (IQR)	0.36 (1.37)	0.26 (1.40)	0.41 (1.37)	0.24^c^
Height for age Z-score, mean ± SD	0.06 ± 1.31	−0.00 ± 1.35	0.26 ± 1.20	0.42^b^
BMI for age Z-score, median (IQR)	0.45 (1.50)	0.21 (1.83)	0.67 (1.18)	0.50^c^
**Child’s household**				
Lives with both parents [*n* (%)]	39 (44)	30 (45)	9 (41)	0.40^d^
Lives with both parents and sibling(s) [*n* (%)]	39 (44)	29 (43)	10 (45)	
Lives with both patents, sibling(s) and others [*n* (%)]	4 (4)	3 (4)	1 (5)	
Lives with both parents and others [*n* (%)]	2 (2)	1 (1)	1 (5)	
Lives with father and sibling(s) [*n* (%)]	1 (1)	0 (0)	1 (5)	
Lives with mother [*n* (%)]	4 (4)	4 (6)	0 (0)	
**Does the child have any food intolerance or allergy?**				
Yes [*n* (%)]	6 (7)	5 (7)	1 (5)	1.00^e^
No [*n* (%)]	83 (93)	62 (93)	21 (95)	
Parent’s age (years), median (IQR)	36.0 (7.0)	37.0 (6.0)	35.0 (8.0)	0.12^b^
**Parent gender**				
Female [*n* (%)]	83 (93)	62 (93)	21 (95)	1.00^e^
Male [*n* (%)]	6 (7)	5 (7)	1 (5)	
**Parent’s education**				
Middle school [*n* (%)]	7 (8)	5 (7)	2 (9)	0.49^a^
High school [*n* (%)]	28 (31)	19 (28)	9 (41)	
University [*n* (%)]	54 (61)	43 (64)	11 (50)	
Parent’s BMI*				
Mean ± SD	24.74 ± 4.34	24.45 ± 4.11	25.61 ± 4.98	0.28^b^
Underweight [*n* (%)]	3 (3)	3 (4)	0 (0)	0.75^d^
Normal weight [*n* (%)]	46 (52)	34 (51)	12 (55)	
Overweight [*n* (%)]	30 (34)	23 (34)	7 (32)	
Obese [*n* (%)]	9 (10)	6 (9)	3 (14)	

BMI, body mass index; IQR, interquartile range; SD, standard deviation. ^a^Statistical test used: Pearson’s Chi-squared test; ^b^Statistical test used: Students *t*-test; ^c^Statistical test used: Mann–Whitney test; ^d^Statistical test used: Chi-squared test by Monte Carlo simulation. ^e^Statistical test used: Fisher’s exact test. *Missing information: 1 parent did not have information on BMI. A *p*-value of less than 0.05 was considered statistically significant.

**TABLE 3:** Whole day dietary intake of macro and micronutrients from each food group evaluated of participants with a high reporting to theSmartKidsDiet24 (*n* = 22).

	Children aged ≤3 years old				Children aged ≥4 years old			
	Added sugar	Fruits	Vegetables	Legumes	Added sugar	Fruit	Vegetables	Legumes
Portions	1.2 ± 1.0	2.3 ± 1.2	2.0 (2.0)	0.2 (1.2)	1.6 ± 0.8	1.5 ± 1.0	1.1 (1.3)	0.1 (0.1)
Energy (kcal)	143.8 (406.7)	146.1 ± 76.2	52.5 (62.7)	13.3 (128.4)	481.0 (456.8)	101.6 ± 67.4	39.4 (35.1)	9.0 (16.3)
Protein (g)	2.7 (8.5)	1.6 ± 1.0	2.3 (2.4)	0.9 (11.5)	7.4 (8.9)	1.0 ± 0.7	1.1 (1.3)	0.6 (1.1)
Carbohydrates (g)	19.0 (77.8)	29.9 ± 15.5	5.9 (7.5)	1.8 (6.3)	71.4 (75.5)	21.1 ± 14.2	4.6 (4.7)	1.0 (2.1)
Sugars (g)	14.0 (28.0)	28.9 ± 15.1	2.0 (2.7)	0.2 (1.2)	41.1 (50.2)	20.4 ± 13.8	1.5 (2.2)	0.1 (0.2)
Dietary fiber (g)	0.8 (3.2)	5.0 ± 2.6	1.9 (2.2)	0.5 (1.3)	3.9 (7.8)	3.1 ± 1.9	1.1 (1.3)	0.3 (0.6)
Fats (g)	4.1 (6.6)	0.9 ± 0.5	1.1 (2.3)	0.1 (5.9)	14.4 (15.3)	0.6 ± 0.4	1.1 (1.7)	0.1 (0.5)
MUFA (g)	1.3 (2.8)	0.1 (0.1)	0.2 (1.7)	0.0 (0.5)	2.9 (6.7)	0.0 (0.1)	0.6 (1.2)	0.0 (0.2)
PUFA (g)	0.8 (0.6)	0.3 ± 0.2	0.3 (0.4)	0.0 (0.3)	0.9 (2.0)	0.2 ± 0.1	0.1 (0.2)	0.0 (0.1)
SFA (g)	1.7 (2.8)	0.1 (0.1)	1.1 (0.4)	0.0 (0.8)	5.4 (8.2)	0.1 (0.1)	0.2 (0.3)	0.0 (0.1)
*Trans* fats (g)	0.1 (0.3)	0.0 (0.0)	0.0 (0.0)	0.0 (0.0)	0.1 (0.8)	0.0 (0.0)	0.0 (0.0)	0.0 (0.0)
Calcium (mg)	57.2 (63.8)	29.3 ± 20.9	36.8 (55.0)	5.1 (27.5)	89.3 (86.2)	13.4 ± 9.1	24.3 (24.3)	3.3 (5.8)
Potassium (mg)	35.5 (148.3)	493.7 ± 269.6	295.0 (359.2)	44.6 (101.3)	187.5 (271.2)	338.2 ± 231.2	165.0 (139.5)	25.0 (41.3)
Sodium (mg)	142.3 (189.4)	15.0 (6.3)	357.3 (388.3)	19.8 (321.3)	155.6 (198.7)	8.0 (7.5)	198.0 (262.1)	18.5 (31.3)
Vitamin B12 (mg)	0.0 (0.2)	0.0 (0.00)	0.0 (0.0)	0.0 (0.0)	0.0 (0.0)	0.0 (0.0)	0.0 (0.0)	0.0 (0.0)
Vitamin C (mg)	0.0 (3.2)	38.9 (49.8)	14.1 (22.4)	0.0 (2.8)	0.0 (2.0)	8.8 (18.3)	6.5 (10.9)	0.0 (1.6)
Vitamin D (mg)	0.0 (0.6)	0.0 (0.0)	0.0 (0.0)	0.0 (0.0)	0.0 (0.1)	0.0 (0.0)	0.0 (0.1)	0.0 (0.0)

G, grams; Kcal, kilocalories; Mg, milligrams; MUFA, monounsaturated fatty acids; PUFA, polyunsaturated fatty acids; SFA, saturated fatty acids. Values are presented as mean ± SD or median (IQR), according to the distribution of data.

**TABLE 4:** Dietary intake of macro and micronutrients from added sugar foods discriminated per meal of participants aged ≤3 years old (*n* = 7) and aged ≥4 years old (*n* = 15) with a high reporting to theSmartKidsDiet24.

	Added sugar foods											
	Children aged ≤3 years old						Children aged ≥4 years old					
	Breakfast	Morning snack	Lunch	Afternoon snack	Dinner	Night snack	Breakfast	Morning snack	Lunch	Afternoon snack	Dinner	Night snack
Portions	0.0 (0.6)	0.0 (0.5)	0.0 (0.1)	0.5 ± 0.3	0.0 (0.3)	0.0 (0.0)	0.5 (0.8)	0.0 (0.4)	0.0 (0.0)	0.6 ± 0.4	0.0 (0.0)	0.0 (0.0)
Energy (kcal)	0.0 (226.3)	0.0 (209.0)	0.0 (1.6)	39.5 (105.2)	0.0 (14.3)	0.0 (0.0)	0.0 (367.8)	0.0 (163.5)	0.0 (0.0)	91.7 (297.5)	0.0 (0.0)	0.0 (0.0)
Protein (g)	0.0 (4.7)	0.0 (4.2)	0.0 (0.0)	1.9 (2.7)	0.0 (0.0)	0.0 (0.0)	3.0 (7.8)	0.0 (3.2)	0.0 (0.0)	2.6 (3.2)	0.0 (0.0)	0.0 (0.0)
Carbohydrates (g)	0.0 (47.0)	0.0 (28.8)	0.0 (0.4)	5.4 (14.7)	0.0 (3.5)	0.0 (0.0)	39.0 (62.5)	0.0 (27.0)	0.0 (0.0)	16.7 (36.2)	0.0 (0.0)	0.0 (0.0)
Sugars (g)	0.0 (16.2)	0.0 (10.8)	0.0 (0.4)	2.3 (4.2)	0.0 (3.4)	0.0 (0.0)	22.3 (34.7)	0.0 (11.7)	0.0 (0.0)	8.6 (32.0)	0.0 (0.0)	0.0 (0.0)
Dietary fiber (g)	0.0 (2.5)	0.0 (7.3)	0.0 (0.0)	0.0 (0.8)	0.0 (0.0)	0.0 (0.0)	1.4 (2.4)	0.0 (5.3)	0.0 (0.0)	0.7 (0.9)	0.0 (0.0)	0.0 (0.0)
Fats (g)	0.0 (1.6)	0.0 (1.1)	0.0 (0.0)	0.6 (4.1)	0.0 (0.0)	0.0 (0.0)	5.0 (9.6)	0.0 (2.8)	0.0 (0.0)	1.5 (13.4)	0.0 (0.0)	0.0 (0.0)
MUFA (g)	0.0 (0.0)	0.0 (3.7)	0.0 (0.0)	0.2 (1.3)	0.0 (0.0)	0.0 (0.0)	0.1 (2.7)	0.0 (2.4)	0.0 (0.0)	0.3 (3.4)	0.0 (0.0)	0.0 (0.0)
PUFA (g)	0.0 (0.0)	0.0 (1.0)	0.0 (0.0)	0.0 (0.6)	0.0 (0.0)	0.0 (0.0)	0.0 (1.1)	0.0 (0.0)	0.0 (0.0)	0.1 (0.6)	0.0 (0.0)	0.0 (0.0)
SFA (g)	0.0 (0.4)	0.0 (1.0)	0.0 (0.0)	0.2 (1.7)	0.0 (0.0)	0.0 (0.0)	0.9 (4.1)	0.0 (0.4)	0.0 (0.0)	0.6 (7.1)	0.0 (0.0)	0.0 (0.0)
*Trans* fats (g)	0.0 (0.0)	0.0 (1.0)	0.0 (0.0)	0.0 (0.1)	0.0 (0.0)	0.0 (0.0)	0.0 (0.1)	0.0 (0.1)	0.0 (0.0)	0.0 (0.1)	0.0 (0.0)	0.0 (0.0)
Calcium (mg)	0.0 (0.0)	0.0 (0.1)	0.0 (0.0)	21.7 (54.0)	0.0 (0.0)	0.0 (0.0)	10.5 (52.0)	0.0 (0.0)	0.0 (0.0)	52.0 (73.7)	0.0 (0.0)	0.0 (0.0)
Potassium (mg)	0.0 (0.0)	0.0 (23.8)	0.0 (0.0)	28.3 (80.0)	0.0 (0.0)	0.0 (0.0)	42.3 (150.8)	0.0 (60.0)	0.0 (0.0)	56.7 (149.7)	0.0 (0.0)	0.0 (0.0)
Sodium (mg)	0.0 (0.0)	0.0 (37.5)	0.0 (4.8)	34.8 (83.1)	0.0 (42.8)	0.0 (0.0)	8.0 (74.3)	0.0 (80.0)	0.0 (0.0)	40.0 (50.9)	0.0 (0.0)	0.0 (0.0)
Vitamin B12 (mg)	0.0 (0.0)	0.0 (0.0)	0.0 (0.0)	0.0 (0.0)	0.0 (0.0)	0.0 (0.0)	0.0 (0.0)	0.0 (0.0)	0.0 (0.0)	0.0 (0.0)	0.0 (0.0)	0.0 (0.0)
Vitamin C (mg)	0.0 (0.0)	0.0 (0.0)	0.0 (0.0)	0.0 (0.0)	0.0 (0.0)	0.0 (0.0)	0.0 (0.0)	0.0 (0.0)	0.0 (0.0)	0.0 (0.5)	0.0 (0.0)	0.0 (0.0)
Vitamin D (mg)	0.0 (0.0)	0.0 (0.0)	0.0 (0.0)	0.0 (0.0)	0.0 (0.0)	0.0 (0.0)	0.0 (0.0)	0.0 (0.0)	0.0 (0.0)	0.0 (0.0)	0.0 (0.0)	0.0 (0.0)

G, grams; Kcal, kilocalories; Mg, milligrams; MUFA, monounsaturated fatty acids; PUFA, polyunsaturated fatty acids; SFA, saturated fatty acids. Values are presented as mean ± SD or median (IQR), according to the distribution of data.

**TABLE 5:** Dietary intake of macro and micronutrients from fruit discriminated per meal of participants aged ≤3 years old (*n* = 7) and aged ≥4 years old with a high reporting to theSmartKidsDiet24 (*n* = 15).

	Fruit											
	Children aged ≤3 years old						Children aged ≥4 years old					
	Breakfast	Morning snack	Lunch	Afternoon snack	Dinner	Night snack	Breakfast	Morning snack	Lunch	Afternoon snack	Dinner	Night snack
Portions	0.0 (0.5)	0.8 (0.7)	1.0 (1.0)	0.0 (0.6)	0.5 (0.8)	0.0 (0.0)	0.0 (0.0)	0.0 (0.1)	0.4 (0.8)	0.1 (0.4)	0.2 (0.6)	0.0 (0.0)
Energy (kcal)	0.0 (26.5)	52.0 (29.8)	46.0 (75.5)	0.0 (42.1)	35.3 (30.9)	0.0 (0.0)	0.0 (0.0)	0.0 (0.0)	23.5 (40.9)	0.0 (35.4)	10.3 (52.0)	0.0 (0.0)
Protein (g)	0.0 (0.2)	0.4 (0.6)	0.9 (1.0)	0.0 (0.2)	0.2 (0.6)	0.0 (0.0)	0.0 (0.0)	0.0 (0.0)	0.1 (0.5)	0.0 (0.3)	0.1 (0.4)	0.0 (0.0)
Carbohydrates (g)	0.0 (5.6)	10.9 (5.5)	8.2 ± 7.2	0.0 (8.8)	7.1 (5.9)	0.0 (0.0)	0.0 (0.0)	0.0 (0.0)	5.8 ± 5.5	0.0 (7.3)	2.2 (10.9)	0.0 (0.0)
Sugars (g)	0.0 (5.6)	9.8 (6.0)	7.8 ± 6.8	0.0 (8.5)	7.1 (5.9)	0.0 (0.0)	0.0 (0.0)	0.0 (0.0)	5.6 ± 5.2	0.0 (7.2)	1.2 (9.8)	0.0 (0.0)
Dietary fiber (g)	0.0 (1.1)	1.6 (1.2)	1.4 ± 1.2	0.0 (1.4)	1.1 (1.5)	0.0 (0.0)	0.0 (0.0)	0.0 (0.0)	0.9 ± 0.9	0.0 (1.4)	0.3 (1.5)	0.0 (0.0)
Fats (g)	0.0 (0.2)	0.3 (0.3)	0.2 ± 0.2	0.0 (0.3)	0.2 (0.2)	0.0 (0.0)	0.0 (0.0)	0.0 (0.0)	0.2 ± 0.2	0.0 (0.3)	0.1 (0.3)	0.0 (0.0)
MUFA (g)	0.0 (0.0)	0.0 (0.1)	0.0 (0.1)	0.0 (0.0)	0.0 (0.1)	0.0 (0.0)	0.0 (0.0)	0.0 (0.0)	0.0 (0.1)	0.0 (0.0)	0.0 (0.0)	0.0 (0.0)
PUFA (g)	0.0 (0.1)	0.1 (0.1)	0.1 ± 0.1	0.0 (0.1)	0.1 (0.1)	0.0 (0.0)	0.0 (0.0)	0.0 (0.0)	0.1 ± 0.1	0.0 (0.1)	0.0 (0.1)	0.0 (0.0)
SFA (g)	0.0 (0.0)	0.1 (0.1)	0.0 (0.1)	0.0 (0.1)	0.0 (0.1)	0.0 (0.0)	0.0 (0.0)	0.0 (0.0)	0.0 (0.1)	0.0 (0.1)	0.0 (0.1)	0.0 (0.0)
*Trans* fats (g)	0.0 (0.0)	0.0 (0.0)	0.0 (0.0)	0.0 (0.0)	0.0 (0.0)	0.0 (0.0)	0.0 (0.0)	0.0 (0.0)	0.0 (0.0)	0.0 (0.0)	0.0 (0.0)	0.0 (0.0)
Calcium (mg)	0.0 (5.5)	5.4 (9.8)	8.5 (14.9)	0.0 (4.2)	3.0 (11.6)	0.0 (0.0)	0.0 (0.0)	0.0 (0.0)	2.8 (7.3)	0.0 (4.1)	0.9 (5.0)	0.0 (0.0)
Potassium (mg)	0.0 (80.0)	140.0 (110.0)	200.0 (280.0)	0.0 (121.3)	112.5 (133.8)	0.0 (0.0)	0.0 (0.0)	0.0 (0.0)	65.0 (140.0)	0.0 (107.8)	35.8 (175.0)	0.0 (0.0)
Sodium (mg)	0.0 (2.0)	4.5 (5.0)	3.6 ± 3.0	0.0 (4.0)	3.0 (5.8)	0.0 (0.0)	0.0 (0.0)	0.0 (0.0)	3.2 ± 3.0	0.0 (3.8)	1.0 (4.0)	0.0 (0.0)
Vitamin B12 (mg)	0.0 (0.0)	0.0 (0.0)	0.0 (0.0)	0.0 (0.0)	0.0 (0.0)	0.0 (0.0)	0.0 (0.0)	0.0 (0.0)	0.0 (0.0)	0.0 (0.0)	0.0 (0.0)	0.0 (0.0)
Vitamin C (mg)	0.0 (6.0)	4.5 (2.5)	8.0 (21.4)	0.0 (3.9)	3.0 (15.4)	0.0 (0.0)	0.0 (0.0)	0.0 (0.0)	2.0 (4.5)	0.0 (7.8)	1.0 (3.8)	0.0 (0.0)
Vitamin D (mg)	0.0 (0.0)	0.0 (0.0)	0.0 (0.0)	0.0 (0.0)	0.0 (0.0)	0.0 (0.0)	0.0 (0.0)	0.0 (0.0)	0.0 (0.0)	0.0 (0.0)	0.0 (0.0)	0.0 (0.0)

G, grams; Kcal, kilocalories; Mg, milligrams; MUFA, monounsaturated fatty acids; PUFA, polyunsaturated fatty acids; SFA, saturated fatty acids. Values are presented as mean ± SD or median (IQR), according to the distribution of data.

**TABLE 6:** Dietary intake of macro and micronutrients from vegetables discriminated per meal of participants aged ≤3 years old (*n* = 7) and aged ≥4 years old (*n* = 15) with a high reporting to the theSmartKidsDiet24.

	Vegetables											
	Children aged ≤3 years old						Children aged ≥4 years old					
	Breakfast	Morning snack	Lunch	Afternoon snack	Dinner	Night snack	Breakfast	Morning snack	Lunch	Afternoon snack	Dinner	Night snack
Portions	0.0 (0.0)	0.0 (0.0)	1.0 (1.8)	0.0 (0.0)	0.8 (0.9)	0.0 (0.0)	0.0 (0.0)	0.0 (0.0)	0.7 (1.1)	0.0 (0.0)	0.5 (1.0)	0.0 (0.0)
Energy (kcal)	0.0 (0.0)	0.0 (0.0)	30.3 (39.5)	0.0 (0.0)	18.3 (32.4)	0.0 (0.0)	0.0 (0.0)	0.0 (0.0)	17.6 (36.7)	0.0 (0.0)	16.5 (24.8)	0.0 (0.0)
Protein (g)	0.0 (0.0)	0.0 (0.0)	1.0 (1.8)	0.0 (0.0)	0.7 (0.7)	0.0 (0.0)	0.0 (0.0)	0.0 (0.0)	0.7 (1.1)	0.0 (0.0)	0.6 (1.0)	0.0 (0.0)
Carbohydrates (g)	0.0 (0.0)	0.0 (0.0)	3.2 (4.9)	0.0 (0.0)	2.2 (4.4)	0.0 (0.0)	0.0 (0.0)	0.0 (0.0)	2.5 (5.0)	0.0 (0.0)	2.1 (3.1)	0.0 (0.0)
Sugars (g)	0.0 (0.0)	0.0 (0.0)	0.8 (1.7)	0.0 (0.0)	1.0 (1.8)	0.0 (0.0)	0.0 (0.0)	0.0 (0.0)	0.8 (1.4)	0.0 (0.0)	0.6 (1.3)	0.0 (0.0)
Dietary fiber (g)	0.0 (0.0)	0.0 (0.0)	1.0 (1.5)	0.0 (0.0)	0.7 (0.8)	0.0 (0.0)	0.0 (0.0)	0.0 (0.0)	0.6 (1.4)	0.0 (0.0)	0.7 (0.9)	0.0 (0.0)
Fats (g)	0.0 (0.0)	0.0 (0.0)	0.6 (1.6)	0.0 (0.0)	0.4 (0.6)	0.0 (0.0)	0.0 (0.0)	0.0 (0.0)	0.3 (1.2)	0.0 (0.0)	0.5 (1.1)	0.0 (0.0)
MUFA (g)	0.0 (0.0)	0.0 (0.0)	0.0 (1.1)	0.0 (0.0)	0.1 (0.6)	0.0 (0.0)	0.0 (0.0)	0.0 (0.0)	0.1 (0.8)	0.0 (0.0)	0.3 (0.7)	0.0 (0.0)
PUFA (g)	0.0 (0.0)	0.0 (0.0)	0.2 (0.3)	0.0 (0.0)	0.1 (0.1)	0.0 (0.0)	0.0 (0.0)	0.0 (0.0)	0.1 (0.1)	0.0 (0.0)	0.1 (0.1)	0.0 (0.0)
SFA (g)	0.0 (0.0)	0.0 (0.0)	0.1 (0.2)	0.0 (0.0)	0.1 (0.1)	0.0 (0.0)	0.0 (0.0)	0.0 (0.0)	0.1 (0.2)	0.0 (0.0)	0.1 (0.2)	0.0 (0.0)
*Trans* fats (g)	0.0 (0.0)	0.0 (0.0)	0.0 (0.0)	0.0 (0.0)	0.0 (0.0)	0.0 (0.0)	0.0 (0.0)	0.0 (0.0)	0.0 (0.0)	0.0 (0.0)	0.0 (0.0)	0.0 (0.0)
Calcium (mg)	0.0 (0.0)	0.0 (0.0)	20.3 (42.1)	0.0 (0.0)	15.8 (8.5)	0.0 (0.0)	0.0 (0.0)	0.0 (0.0)	13.2 (24.8)	0.0 (0.0)	10.3 (24.0)	0.0 (0.0)
Potassium (mg)	0.0 (0.0)	0.0 (0.0)	115.0 (262.5)	0.0 (0.0)	113.8 (130.0)	0.0 (0.0)	0.0 (0.0)	0.0 (0.0)	102.5 (155.7)	0.0 (0.0)	77.5 (147.9)	0.0 (0.0)
Sodium (mg)	0.0 (0.0)	0.0 (0.0)	228.8 (325.8)	0.0 (0.0)	125.0 (214.8)	0.0 (0.0)	0.0 (0.0)	0.0 (0.0)	126.2 (243.3)	0.0 (0.0)	110.0 (193.5)	0.0 (0.0)
Vitamin B12 (mg)	0.0 (0.0)	0.0 (0.0)	0.0 (0.0)	0.0 (0.0)	0.0 (0.0)	0.0 (0.0)	0.0 (0.0)	0.0 (0.0)	0.0 (0.0)	0.0 (0.0)	0.0 (0.0)	0.0 (0.0)
Vitamin C (mg)	0.0 (0.0)	0.0 (0.0)	5.9 (11.9)	0.0 (0.0)	4.9 (11.9)	0.0 (0.0)	0.0 (0.0)	0.0 (0.0)	4.7 (7.1)	0.0 (0.0)	1.9 (7.3)	0.0 (0.0)
Vitamin D (mg)	0.0 (0.0)	0.0 (0.0)	0.0 (0.0)	0.0 (0.0)	0.0 (0.0)	0.0 (0.0)	0.0 (0.0)	0.0 (0.0)	0.0 (0.0)	0.0 (0.0)	0.0 (0.0)	0.0 (0.0)

G, grams; Kcal, kilocalories; Mg, milligrams; MUFA, monounsaturated fatty acids; PUFA, polyunsaturated fatty acids; SFA, saturated fatty acids. Values are presented as median (IQR).

**TABLE 7:** Dietary intake of macro and micronutrients from legumes discriminated per meal of participants aged ≤3 years old (*n* = 7) and aged ≥4 years old (*n* = 15) with a high reporting to theSmartKidsDiet24.

	Legumes											
	Children aged ≤3 years old						Children aged ≥4 years old					
	Breakfast	Morning snack	Lunch	Afternoon snack	Dinner	Night snack	Breakfast	Morning snack	Lunch	Afternoon snack	Dinner	Night snack
Portions	0.0 (0.0)	0.0 (0.0)	0.2 (0.3)	0.0 (0.0)	0.0 (0.6)	0.0 (0.0)	0.0 (0.0)	0.0 (0.0)	0.0 (0.1)	0.0 (0.0)	0.0 (0.1)	0.0 (0.0)
Energy (kcal)	0.0 (0.0)	0.0 (0.0)	13.3 (33.4)	0.0 (0.0)	0.0 (74.8)	0.0 (0.0)	0.0 (0.0)	0.0 (0.0)	0.0 (9.3)	0.0 (0.0)	0.0 (16.3)	0.0 (0.0)
Protein (g)	0.0 (0.0)	0.0 (0.0)	0.7 (2.8)	0.0 (0.0)	0.0 (5.5)	0.0 (0.0)	0.0 (0.0)	0.0 (0.0)	0.0 (0.2)	0.0 (0.0)	0.0 (1.1)	0.0 (0.0)
Carbohydrates (g)	0.0 (0.0)	0.0 (0.0)	1.3 (1.9)	0.0 (0.0)	0.0 (5.1)	0.0 (0.0)	0.0 (0.0)	0.0 (0.0)	0.0 (1.4)	0.0 (0.0)	0.0 (2.1)	0.0 (0.0)
Sugars (g)	0.0 (0.0)	0.0 (0.0)	0.2 (0.3)	0.0 (0.0)	0.0 (0.9)	0.0 (0.0)	0.0 (0.0)	0.0 (0.0)	0.0 (0.1)	0.0 (0.0)	0.0 (0.1)	0.0 (0.0)
Dietary fiber (g)	0.0 (0.0)	0.0 (0.0)	0.0 (0.4)	0.0 (0.0)	0.0 (1.1)	0.0 (0.0)	0.0 (0.0)	0.0 (0.0)	0.0 (0.2)	0.0 (0.0)	0.0 (0.6)	0.0 (0.0)
Fats (g)	0.0 (0.0)	0.0 (0.0)	0.1 (1.8)	0.0 (0.0)	0.0 (3.1)	0.0 (0.0)	0.0 (0.0)	0.0 (0.0)	0.0 (0.2)	0.0 (0.0)	0.0 (0.3)	0.0 (0.0)
MUFA (g)	0.0 (0.0)	0.0 (0.0)	0.0 (0.1)	0.0 (0.0)	0.0 (0.5)	0.0 (0.0)	0.0 (0.0)	0.0 (0.0)	0.0 (0.1)	0.0 (0.0)	0.0 (0.1)	0.0 (0.0)
PUFA (g)	0.0 (0.0)	0.0 (0.0)	0.0 (0.1)	0.0 (0.0)	0.0 (0.2)	0.0 (0.0)	0.0 (0.0)	0.0 (0.0)	0.0 (0.1)	0.0 (0.0)	0.0 (0.1)	0.0 (0.0)
SFA (g)	0.0 (0.0)	0.0 (0.0)	0.0 (0.2)	0.0 (0.0)	0.0 (0.5)	0.0 (0.0)	0.0 (0.0)	0.0 (0.0)	0.0 (0.0)	0.0 (0.0)	0.0 (0.0)	0.0 (0.0)
*Trans* fats (g)	0.0 (0.0)	0.0 (0.0)	0.0 (0.0)	0.0 (0.0)	0.0 (0.0)	0.0 (0.0)	0.0 (0.0)	0.0 (0.0)	0.0 (0.0)	0.0 (0.0)	0.0 (0.0)	0.0 (0.0)
Calcium (mg)	0.0 (0.0)	0.0 (0.0)	0.0 (6.5)	0.0 (0.0)	0.0 (9.0)	0.0 (0.0)	0.0 (0.0)	0.0 (0.0)	0.0 (2.8)	0.0 (0.0)	0.0 (5.8)	0.0 (0.0)
Potassium (mg)	0.0 (0.0)	0.0 (0.0)	0.0 (42.2)	0.0 (0.0)	0.0 (41.3)	0.0 (0.0)	0.0 (0.0)	0.0 (0.0)	0.0 (16.7)	0.0 (0.0)	0.0 (33.8)	0.0 (0.0)
Sodium (mg)	0.0 (0.0)	0.0 (0.0)	0.9 (19.8)	0.0 (0.0)	0.0 (319.7)	0.0 (0.0)	0.0 (0.0)	0.0 (0.0)	0.0 (12.5)	0.0 (0.0)	0.0 (31.3)	0.0 (0.0)
Vitamin B12 (mg)	0.0 (0.0)	0.0 (0.0)	0.0 (0.0)	0.0 (0.0)	0.0 (0.0)	0.0 (0.0)	0.0 (0.0)	0.0 (0.0)	0.0 (0.0)	0.0 (0.0)	0.0 (0.0)	0.0 (0.0)
Vitamin C (mg)	0.0 (0.00)	0.0 (0.0)	0.0 (1.6)	0.0 (0.0)	0.0 (0.8)	0.0 (0.0)	0.0 (0.0)	0.0 (0.0)	0.0 (0.1)	0.0 (0.0)	0.0 (0.0)	0.0 (0.0)
Vitamin D (mg)	0.0 (0.0)	0.0 (0.0)	0.0 (0.0)	0.0 (0.0)	0.0 (0.0)	0.0 (0.0)	0.0 (0.0)	0.0 (0.0)	0.0 (0.0)	0.0 (0.0)	0.0 (0.0)	0.0 (0.0)

G, grams; Kcal, kilocalories; Mg, milligrams; MUFA, monounsaturated fatty acids; PUFA, polyunsaturated fatty acids; SFA, saturated fatty acids. Values are presented as median (IQR).

## 4. Discussion

In our cohort, children showed inadequate consumption of the four key food groups studied. In fact, children showed a higher intake of added sugar foods than vegetables and legumes. Of interest, consumption was only reported in the presence of parents or if parents were sure about their children’s dietary intake, which may indicate that this data could still be undervalued. Moreover, the method used to estimate food portions consumed by the child implies learning a method unknown to the parents (using the child’s hand to estimate portions) that may not have been fully assimilated at this early stage in the trial.

The contribution of free sugars to the total energy intake of European children has already been reported to be higher than recommendations (16). This is no surprise, as the preference for sweet taste is a universal characteristic of humans (17, 18). Innate dietary preferences in childhood reflect our basic biology, which predisposes to the preference of sweet foods and the avoidance of bitter-tasting items such as green leafy vegetables (19). This is in line with our findings, as we observed a higher intake of added sugar foods compared to vegetables. This biological predisposition to preferer certain foods over others makes it difficult for parents do effectively guide their children to make healthier choices. Moreover, palatable foods, such as added sugar foods, interfere with normal appetite regulation, that is, with the complex interplay between hunger and satiety signals (20). It has been suggested that excessive consumption of sugar is facilitated by a shift in a hunger-satiety continuum that leads an individual to feel hungry for sugar despite a lack of an actual energy need, and reaches satiety later, ultimately promoting the maintenance of its consumption (21). Eating in the absence of hunger has been shown to be positively associated with an increased weight status among young children (22). Overall, sweetness is a potent stimulus for humans of all ages, and this attraction for sweet foods and beverages may stimulate overeating and induce weight gain in the long term (23). Moreover, school food environments have been shown to affect dietary behaviors of school children, including the consumption of sugar-sweetened beverages and, in fact, children’s homes were significant sources of sugar-sweetened beverages consumed at school (24). Intervening in the food environment is, therefore, critical to improve children diets, in both school and community settings (25). Among pre-school children, home availability of sugar-enriched foods were shown to be positively associated with a sweets-and-treats dietary pattern (a dietary pattern high in foods such as sweet biscuits, chocolate, ice cream) and inversely associated with the health-conscious pattern (high in foods such as nuts, natural yogurt, and berries) in the children (26). This highlights the crucial role of parents in managing children’s food environment at home and, consequently, highlights the need for parent-targeted interventions in this population.

In Portugal, 16% of children aged 6–8 years old were reported to consume sweet snacks (cookies/biscuits, sweets, cakes, and doughnuts) four or more times a week, and 80% eat these foods up to three times a week (4). Moreover, 14% of children drink sweetened beverages four or more times a week, and 71% drink these up to three times a week (4). Still, regarding the consumption of cakes and sweets, 65% of children of Portuguese 4-year-old children were reported to consume these foods at least once a day (27). On this subject, the WHO recommends reducing the intake of free sugars to less than 10% of total energy intake both for adults and children (28). The fact that in our sample over 80% of children exceeded this recommendation at baseline, and as early-life experiences concerning taste and flavor are relevant for the promotion of healthy dietary patterns later in life (29), further justifies interventions in this field.

In our cohort, fruit intake was higher than vegetable intake. Nationally, daily consumption of fruits has been reported more frequent (63%) than vegetable soup (57%) (4). Moreover, previous data reports 92% of Portuguese 4-year-old children consume soup at least once a day, 45% consume vegetables daily, cooked or in salads, 86% consume fresh fruit daily and 59% consume it two or more times a day (27). Of interest, parent-targeted interventions have been shown to result in significant increase in fruit and vegetable intake in children (30). Increasing parent’s knowledge is relevant as pressure-to-eat is counterproductive and can have negative effects (31). Regarding strategies for changing children’s eating behaviors, evidence suggests that hands-on approaches such as gardening and cooking, as well as providing children with free, accessible fruits and vegetables may encourage a greater consumption of these foods (32).

In our sample, children’s sodium intake was high, especially considering total dietary intake was not evaluated and, thus, children’s actual daily intake of sodium may be even higher. Having 13.6% of children exceed the recommendation just through four food groups that exclude the recording of key sodium sources, such as processed and fast foods (apart from those that have added sugar) may be a strong indicator of a nutritionally poor dietary pattern. Current WHO recommendation on sodium consumption for adults is 2 g sodium/day (33). This recommendation has already been shown to be largely exceeded by Portuguese adults (34). As for children, WHO states that the recommended maximum level of intake of 2 g/day sodium in adults should be adjusted downward based on the energy requirements of children relative to those of adults (33). In our sample children’s sodium intake was high, which is in line with current evidence that suggests that Portuguese children also have high sodium intake (35). As for potassium, we found that 77.3% of our sample had potassium intakes below the recommended. Although we haven’t considered children’s whole dietary pattern, since vegetables, fruits and legumes are some of the main dietary sources of potassium, these findings support previous national and international data that report that young children do not consume enough potassium (36–38). Similarly, these food groups are among the main dietary sources of fiber and vitamin C. In our sample, 73.3 and 31.8% of children showed low compliance to the fiber and vitamin C intake recommendations, respectively. An adequate fiber intake in children and adolescents might be associated with a lower risk of obesity, constipation, metabolic syndrome, insulin resistance, and high blood pressure (39). Low vitamin C, an essential nutrient that must be obtained through the diet in adequate amounts, is thought to be both a cause and a consequence of various communicable and non-communicable diseases (40). Hence, considering their low intake in our cohort, dietary interventions aimed at increasing the consumption of fiber and vitamin C rich foods are warranted. As expected, all children did not meet their calcium, vitamin B12 and vitamin D intake recommendations, most likely due to the lack of information regarding their consumption of the main dietary sources of these nutrients, such dairy, meats and fish.

This study has several limitations. Collected data was self-reported, making it more vulnerable to errors. As previously mentioned, parents were instructed to register only the foods they were sure the child ate, meaning there may be missing meals/snacks eaten by the kids in the absence of parents. On the other hand, children with a low reporting to the without recordings of at least five meals per day in at least 2 days were excluded from the detailed dietary analysis, although we do not know if they did not have all five meals recorded due to actual missing information on their diet or if they did not eat these meals at all. This is major limitation of our study, as we cannot be sure if the nutrient intakes represent true whole-day dietary intakes. This weaknesses of the app and study protocol could have been solutioned by allowing more than one log-in per child, that is, both parents and other family members/caregivers (when applicable) could register the child’s dietary intake. This may have prevented some missing data on the theSmartKidsDiet24. The app should also have included an option so parents (or others) could specify whether the child did not have the meal in question or if they are not sure because they were not present. This way, some of the considered incomplete theSmartKidsDiet24 reports could have been included in the analysis, if we knew that the child didn’t actually eat and it was not the case of missing data.

## 5. Conclusion

Our findings suggest that the dietary intake of key components of a healthy dietary pattern of Portuguese preschool children is inadequate, with a high consumption of sugary foods and low intake of vegetables and legumes. As current literature on diet in overall health strongly states that dietary patterns rich in processed foods with low nutritional value and high in calories, and low in nutrient dense foods like vegetables and legumes are linked to poor health throughout the life course, the establishment of healthy dietary patterns from a young age is warranted.

Children’s dietary intake was assessed based on parents reports and, thus, represent estimations only, as it remains unknown whether children consumed other non-reported foods. Nevertheless, our results further justify the need for interventions in this field, such as the SmartFeeding4Kids program, designed to be an intervention for parents who want to improve their feeding practices and develop a healthy diet in their young children. There is no doubt that consistent systemic changes are needed to fully address this problematic, namely with regard to the promotion of an environment where the availability and access to healthy foods is improved. Nevertheless, when considering in young children, it is recognized that parent targeted interventions are valuable strategies to promote healthier eating. Mobile apps have the potential to share information in a flexible, easy, and intuitive format in a cost-effective manner. Moreover, they are interesting tools in the fast-paced world we live in, being suitable for time-constrained and overwhelmed parents, as they are easily accessible and can be self-paced.

## Data availability statement

The original contributions presented in this study are included in the article/supplementary material, further inquiries can be directed to the corresponding author.

## Ethics statement

The studies involving human participants were reviewed and approved by the Ethics and Deontology Committee of the Faculty of Psychology, University of Lisbon. Written informed consent to participate in this study was provided by the participants’ legal guardian/next of kin.

## Author contributions

JS, AG, DB, TG, and LB gave scientific support, critically revised the draft, clarified concepts, conceptualized the study, and collected and processed the data. SC and JS interpreted the data, drafted the manuscript, and wrote the manuscript in its final format. All authors contributed to the article and approved the submitted version.
